# How to deal with controversies in the evaluation of medicalization: a case study of PMS and PMDD

**DOI:** 10.1007/s11019-026-10374-y

**Published:** 2026-07-06

**Authors:** Ilvie Prince

**Affiliations:** Institute for Ethics, History and Philosophy of Medicine, Hannover Medical School, Hannover, Germany

**Keywords:** Medicalization, PMS, Pragmatic medicine, Philosophy of medicine

## Abstract

Medicalization has become a key topic in contemporary philosophy of medicine. Discussions about what should be medicalized, what kinds of medicalization are desirable, and how specific cases ought to be evaluated increasingly shape debates about the boundaries and responsibilities of medicine. The medicalization debate is increasingly shaped by pragmatic considerations that draw attention to the complex interactions between mind and body and to the role of social and environmental conditions in shaping health. From this perspective, the value of medical interventions and resources lies in their capacity to contribute to broader goals such as alleviating human suffering and promoting individual and collective well-being. While pragmatic approaches address several shortcomings of stricter, often essentialist frameworks, their existing analytical tools remain limited when it comes to evaluating controversial cases of medicalization. This paper examines these limitations and develops a refined pragmatic framework for distinguishing good from problematic medicalization. Using the case study of the pharmaceutical treatment of behavioral and mood-related symptoms associated with premenstrual syndrome (PMS) and premenstrual dysphoric disorder (PMDD), I propose a two-step approach. This two-step approach acknowledges the complexities of pragmatic medicalization and distinguishes between the potential usefulness of medicines to achieve pragmatic goals and the realization of this potential in actual practice.

## Introduction

In recent years, philosophical debates on medicine have created an interesting tension. On the one hand, there is a critical debate in reaction to the ongoing expansion of disease concepts, medical diagnostics, and interventions into all areas of life, a process also known as medicalization or overmedicalization. Such expansions can give rise to oppressive norms, harm patients, and undermine the professional integrity of medicine. (Hofmann 2025). On the other hand, there is a lively discussion criticizing the conceptual and epistemological ideas that are often used to set these boundaries and guide medical practice. In these debates, philosophers question the often assumed simple equations of pathology with disease (Walker and Rogers 2024; Anjum et al. 2020), dualistic thinking for underestimating the complexity of interactions between the mind and body and the influence of society and the environment on human health (Kukla 2014; Parens 2011; Kaczmarek 2019), and ontological commitments marginalizing single cases (Anjum et al. 2020). They conceptualize disease and health in a pluralistic and context-sensitive way (Kukla 2022; Binney et al. 2024) and medical responsibilities pragmatically along broad goals such as reduction of human suffering (Binney et al. 2024), collective well-being (Kukla 2014), and promoting gender equality and individual well-being (Prince 2023).

Although proponents of pragmatic medical interventions can solve many problems associated with stricter, often essentialist and dualist approaches, which tend to rely on contested concepts or fail to reflect the complexities of human suffering and medical interventions, their existing pragmatic analysis tools also fail to fully articulate the complexities of evaluating medicalization and provide guidance for controversial cases. Existing pragmatic accounts tend to ask whether medicine is useful (in an epistemic, therapeutic, or even social sense); I will argue that we must distinguish potential usefulness from realized usefulness.

In this paper, I will first discuss the shortcomings and lessons from an existing pragmatic approach to distinguishing good from bad medicalization. I focus on this aspect of the medicalization debate by examining whether we should use medical means and practices to intervenen on certain phenomena ((practices of intervention) independently of disease attribution, and how we should handle the risk of creating potentially harmful and oppressive effects. To do so, I will discuss the controversial medicalization of treating primarily behavioral or mood-based symptoms of premenstrual syndrome (PMS) and premenstrual dysphoric disorder (PMDD) with pharmaceutical interventions, demonstrating the shortcomings of existing approaches and highlighting the potential of my account to offer a more fruitful evaluation.^1^ I will propose a two-step approach to analyze whether the medicalization of a condition is good or desirable, as the harm of wrongful medicalization can arise at different stages, especially in controversial cases.

Following the pragmatists’ approach, the first step should be to analyze medicine’s role in the medicalization process and determine whether medicine can contribute to the reduction of human suffering in this specific instance. In other words, this is the question of whether we should medicalize at all. However, I take this to be insufficient to analyze controversial cases such as the PMS/PMDD case. Therefore, if we can establish a potentially beneficial role for medicine, we should evaluate whether this role is being fulfilled properly. This is the often overlooked question of whether a medical practice lives up to our functional and normative expectations.

I argue that there are good normative reasons to medicalize PMS and PMDD. Taking a pragmatic approach to using medical expertise and resources for various conditions and problems is often necessary — and the only responsible solution — in our social environment. However, while acceptable in principle, the medicalization of PMS and PMDD (and probably other cases) involve *practices* that are in tension with the good reasons to medicalize and with legitimate interests of affected individuals. Therefore, I propose transparency requirements that reveal the pragmatic nature of certain controversial cases of medicalization.

## Medicalization: practices and their consequences

While there is no universal definition of medicalization the term was deeply influenced by sociologist Conrad (1992, 2007). Conrad defines medicalization as the process by which X [a condition, set of symptoms, or bodily feature] is defined in medical terms, described using medical language, understood through the adoption of a medical framework, or treated with medical intervention. Although Conrad considered the term medicalization to be value-neutral, medicalization has always been discussed very critically, and continues to be so today. A common term for critically discussed or bad cases of medicalization is overmedicalization (Parens 2011).

While medicalization is often discussed and criticized as one broad practice it actually involves a variety of different practices that can be discussed independently to some extent. The two connected, yet distinct, main practices that are discussed are practices of definition/diagnosis (pathologization) and practices of medical intervention^2^ (Sholl 2017). Prescribing birth control pills is a form of medicalization through intervention without pathologization. After all, we wouldn’t call being fertile and sexually active without wanting children a disease (Prince 2023)^3^. Other cases, such as substance addiction, are more controversial. While some argue that pathologizing substance addiction is inappropriate and unhelpful, others disagree (Walker and Rogers 2024). However, there tends to be general agreement that medical interventions, such as substitution, are helpful. Calling a risk factor such as such as obesity not only risk factor but disease is clearly (controversial) medicalization via practices of definition but not necessarily via practices of intervention, although it can be e.g., when the diagnosis is followed by bariatric surgery or other medical interventions. A cold is an example of medicalization where it becomes very clear that practices of pathologization are not necessarily followed by practices of intervention. Although we diagnose colds and therefore pathologize them, we don’t necessarily actively intervene on the condition with medical means. This is because we assume that otherwise healthy people’s immune systems will defeat the cold without additional intervention, or that home remedies suffice. Medicalization can involve the redefinition of a previously normal part of human variation as pathological but his is neither a necessary starting point nor consequence (Sholl 2017).

The distinction between practices of definition/diagnosis and practices of intervention is not only helpful to discuss more specifically what is problematic in cases of medicalization more specifically, it makes also clear that the different practices involved in medicalization can promote different benefits and harms.

Practices of definition/diagnosis are often discussed based on stigmatization concerns. Defining a condition as disease, as in the case of substance addiction, can remove moral judgement and shame, promote hope for cure but they can also oversimplify, especially when presenting a condition that has complex social and psychological aspects as though it were only a physiological problem (Keane et al. 2022)^4^. Practices of intervention, such as the provision of pharmacological birth control, are often discussed as liberating by offering a feeling of self-control and enhancement. On the other hand, there are worries that that some practices of intervention might have potential oppressive effects by controlling so many aspects in our lives, thereby creating pressure to adjust one’s needs and behavior to fit prevailing standards, promoting the idea that we are objects that need to be fixed, and the responsibility shift of human problems to the medical profession, away from other actors and institutions such as politics that may be better at handling these phenomena (Szasz 1960, 2007; Illich 1976; Zola 1972).

In this paper, I will focus on the latter practices of medicalization (practices of intervention) by discussing when we should use medical means and practices for understanding and treating certain phenomena, and how we should handle the risk of creating or solidifying potentially harmful and oppressive effects by doing so. To limit harmful and stigmatizing effects of overmedicalization, philosophers and ethicists have discussed for many years which criteria should be used to determine whether medicalization is acceptable or even good. I will discuss some of them in the following.

## Medicalization and line drawing

There are different approaches to establishing boundaries for acceptable medical practices i.e. medicalization. While often not made very explicit some line drawings accounts seem to be more concerned with questions of medicalization as practice of intervention, while others are more concerned with medicalization as practice of definition, and others seem to mix these questions.

As I have already indicated, I am primarily interested in the harms of practices of intervention and the question of what kinds of phenomena medicine should intervene on and treat, while remaining agnostic about whether we should call them diseases. Historically, medicine always did more than treating diseases (see Boorse 2016). I will therefore not discuss accounts that draw boundaries based on specific disease definitions.

I will distinguish between two broad types of arguments below. The first group takes a stricter, often essentialist and dualist approach, considering what constitutes (or should constitute) the core of medical practice, medical goals, or central concepts of medicine. The second group uses a pragmatic approach, discussing medicalization in an outcome-oriented manner. They primarily assess its usefulness and “goodness” based on various normative criteria.

The first group that I will present tries to introduce a clear dichotomy between phenomena and practices that are medical in nature and other types of phenomena that should be understood and targeted as social or political issues. Scholars who follow this approach often use essentialist arguments, stating that there is something inherent to medicine that makes it genuinely medical and from which we can derive medical goals and responsibilities. Consequently, actions not called for by medicine’s essence cannot be part of medical practice and are considered bad medicalization or overmedicalization. There are quite a few major problems with these accounts. They raise controversies concerning the scope of medical goals, the right understanding and use of multiple theoretical concepts. The issue that I consider as most problematic is that it is not clear at all why distinguishing good and bad medicalization based on medical goals would reduce the discussed harms of bad medicalization.

Although a detailed description of all accounts discussing the goals of medicine is beyond the scope of this paper, it is important to note that there are many different views of what the right and legitimate goals of medicine are (Hanson & Callahan 2000, Nordenfelt 2001; Pellegrino 2006; Schramme 2025, Boorse 2016) and isn’t something we clearly agree on. There are accounts that argue for an internal morality of medicine (e.g., Pellegrino and Thomasma 1993, Pellegrino 2001) and others that argue for an external morality of medicine (e.g., Veatch 2001; Mathison and Davis 2020). Some argue for multiple goals (e.g., Brody and Miller 1998), others claim that medicine has only one goal, or that the accounts proposing multiple goals can be reduced to one overarching goal of medicine (e.g., Boorse 2016).

Furthermore, the proposed goals vary in scope. Narrow and broad goals face different criticism. Accounts that propose rather narrow goals such as healing as the primary or only goal of medicine have to explain why medicine has historically encompassed activities beyond healing and disease treatment (Boorse 2016), or explain what physicians are doing when they engage in activities that fall outside this narrow conception of medicine but are not considered problematic. Broader goals and moral imperatives present other challenges as they tend to be overly permissive. Take the promotion of health, for example. We see interventions in various societal institutions that aim to achieve this goal. Environmental laws of all kinds, as well as policies such as the requirement to wear seat belts, aim to promote health without being considered medical. Therefore, these broader goals alone are not sufficient to classify something as medical. Similar problems arise with goals such as alleviating people’s suffering and pain (Hofmann 2022).

Many proposed goals require further conceptual work. For example, the intuitively appealing goal of healing raises difficult questions about what should be healed. When we talk about healing this often refers to disease entities. However, debates about what a disease is and how to conceptualize it are as controversial as debates about medical goals. It is also unclear whether this approach would prevent the potential negative consequences of medicalization. Depending on the disease concept, phenomena for which there may be better, less harmful solutions would still be medicalized.

Some scholars try to prevent the debate on the appropriate disease definition by distinguishing medical and non-medical phenomena along the lines of their causes, often referring to physiological or biological causes as deciding factor to put a phenomenon in the realm of medicine (e.g. Daniels 2007). The aim of this strategy is to distribute resources and responsibility using the most suitable solution. According to this approach, this should be achieved by addressing the causes of a problem. For example, a person experiencing depression due to their life circumstances would not be properly treated by medical intervention, say, by altering their brain chemistry, but rather by adjusting social expectations and providing support.

This strategy might sound promising at first, as it calls for responsible stakeholders and recognizes that medical professionals do not necessarily have the expertise to foresee the social consequences of medical interventions, nor the knowledge or expertise to deal with social issues (Callahan 1992; Nordin 2000; Hofmann 2022). However, while it may be true that medical professionals do not have specific expertise in tackling social issues, this often-assumed dichotomy between genuine medical and genuine social issues does not hold up. Such a dichotomy underestimates the complexity of the interactions between mind and body, and environment which impact and constitute what we in practice consider to be targets of medicine (Kukla 2014; Parens 2011; Kaczmarek 2019). Complexity here does not necessarily only mean that there are multiple causes at play such as physiological, cultural, and social causes (as described in the biopsychosocial model by Engel (1977), but that causes can also interact with each other in a way that also influences and alters themselves in the process (Anjum et al. 2020). Therefore phenomena often cannot be divided into their biological, social, and psychological components, since it is precisely the mutual influence of these different areas that constitutes the phenomenon.

Aside from the question whether it is possible to draw such a distinction it is questionable whether this distinction would reach the goal to distribute responsibilities of institutions and stakeholders in a beneficial and less harmful way to provide the most suitable solution to a problem. As discussed in the second section of this paper there are conditions such as substance addiction where it is clear that there are also social causes at play but we know that medical interventions are very successful solutions nonetheless (Parens 2011). Although substance addiction can be addressed through psychotherapy, which teaches healthier coping mechanisms, and through preventive political measures that restrict access to addictive substances, long-term psychotherapy is often not possible without first undergoing medically supervised withdrawal and substitute substance administration. At the same time, social interventions can be in some instances the best solutions for problems that are narrowly defined diseases. For example, classic infectious diseases can often be better controlled by improved hygiene standards and infrastructure.

Whether a medical intervention is the best available solution not only depends on whether it targets the causes, but also on the landscape of alternative solutions, and on the effort, time, money and contextual factors needed for them to succeed. Political changes and social support are often tied to slow and uncertain processes. Shifting the responsibility for those suffering from substance addiction entirely onto political and social institutions would deprive them of a functioning, albeit imperfect, solution and leave them at the mercy of slow societal changes.

Therefore, distinguishing between medical and non-medical phenomena based on their causes is often impossible, and it can also be undesirable, as the consequences may differ from case to case. The ‘social’ and the ‘medical’ are deeply intertwined. Whether intervening on complex phenomena with medical or political and social means is the better option is an empirical question and ultimately depends on feasibility and the benefits and harms of doing so.

Recognizing that conceptual work alone, along with the criteria derived from it, cannot establish satisfactory boundaries, led to a more context-sensitive, pragmatic debate about medicine and its concepts. While a large part of the debate is concerned with pragmatic disease and health concepts (De Vreese 2017; Kukla 2022; Schermer & Binney 2024) there is also work on pragmatic medical goals (Kukla 2014; Jebari 2016; Kaczmarek 2019; Rueda et al. 2021; Prince 2023).

The overall aim of these pragmatic accounts is to promote the acknowledgement that how we conceptualize the world, concepts such as disease and health but also institutions such as medicine have important practical consequences (Schermer & Binney 2024). Pragmatists propose a pluralist approach to do justice to the complexities of medicine and healthcare, where the usefulness and productivity of putting a phenomenon under a disease label or in the hands of medical professionals is highly context-dependent. As I have already indicated, I will not discuss the medicalization as a practice of definition in depth. However, I find it helpful to use an example from the pluralistic conceptualization of disease to demonstrate the context-dependent usefulness that pragmatists have in mind. Consider the controversial question whether deafness should be understood as a disease. Pragmatists would say that in a public project aiming for inclusion and acceptance of sign language in society, calling deafness a disease might not be helpful. Calling deafness a disease in this context might support ideas of deaf people being less capable of communication and different. Understanding deafness as a human variation that comes with different cultures and practices might be more supportive of the project’s strategic goal. At the same time, it can be reasonable to understand deafness as a disease in an insurance setting in order to offer interventions to those who would like to receive it.

Philosophers who are pragmatists on medicalization as a practice of intervention use similar strategies to distinguish good from bad medicalization. They reject the idea that that we can draw a hard line for medicine along the lines of disease treatment (DeVreese 2017) or other medical goals beyond rather broad normatively loaded goals such as the alleviation of human suffering (Schermer & Binney 2024), promotion of collective well-being (Kukla 2014), development of capabilities (Rueda et al. 2021), and promotion of gender equality and individual well-being (Prince 2023). As described above, these goals are too broad to apply solely to the field of medicine. There are multiple other institutions and stakeholders who are also aiming for these goals. However medicine as social institution holds authority over specific social *practices (e.g. trust-based doctor-patient relationship*,* confidentiality)*,* tools (diagnostic tools*,* medical interventions such as pharmacological drugs*,* surgery etc.) and epistemic resources (biomedical knowledge)*, that are different than those of other institutions. A medical intervention can be useful by alleviating suffering, such as by reducing symptoms or curing a disease (Stegenga 2018), or by enabling human flourishing, as with contraceptives (Prince 2023) and gender-affirming care (Kukla 2026). Including a phenomenon under the umbrella of medicine can make us feel understood and cared for as individuals and confer legitimacy. Situating a phenomenon within medicine can also be epistemically useful if existing knowledge or experimental methods help us better understand that phenomenon. These institutional aspects and their usefulness are used by pragmatist as additional qualifying factors to distinguish good and bad medicalization.^5^

Pragmatists therefore argue that the key factor in deciding whether a condition, independent of it being a disease or its causes, should be intervened on medically is whether medicine, given its role, expertise and available tools, can achieve these broader goals (Parens 2011; Kaczmarek 2019), or if we even depend on it to do so (Kukla 2014). However, whether a specific medicalization is good or bad is an empirical question with normative success criteria, as it differs from case to case whether medical resources are helpful or even necessary to achieve our broad goals. It depends on what medicine has to offer and the social context of the condition (Kukla 2014).

To tackle the empirical question concerning specific cases Kaczmarek (2019) proposed four questions to help identify bad medicalization or overmedicalization. She tries to capture socially harmful or oppressive elements of medicalization in her questions by asking:

1. Has X been rightly recognised as a problem?

- Does X cause or significantly increase the risk of considerable physical or mental discomfort, suffering, impairments or death?

2. Does recognising X as a problem not result from unfounded, exaggerated social expectations?

- Is recognising X as a problem not an example of undue limitation of diversity of individuals for the sake of normalization? (Kaczmarek 2019, 122–123)”.

Two further questions address the issue of whether medical institutions, their epistemic resources and their tools are the best solution, or even helpful at all:

3. Does medicine provide the most appropriate methods for understanding X and its causes?^6^

- At what level (e.g., molecular, mental, social, multiple levels combined) do the primary causes of X occur?
- Are there alternative, non-medical and more appropriate ways to understand X and its causes?

4. Does the medicalization of X ensure the most effective and safest methods of resolution?

- Are there alternative, non-medical and more effective ways to solve X or its causes?
- Does the medicalization of X do more harm than good?” (Kaczmarek 2019, p. 123).

It is important to point out that the question asking for causes in Kaczmareks account doesn’t aim for a cause-based evaluation of the medicalization. This question tests for the usefulness of medicine’s explanatory resources.

Pragmatist accounts have many advantages over essentialist or even dualist accounts by giving a more nuanced view of the *usefulness* medical resources and by shedding light on the institutional, societal role of medicine. However, pragmatist also have to acknowledge that the question whether a medicalization is useful with respect to the strategic goals they have in mind, can be quite controversial due to the normative, subjective dimension of these goals (Kukla 2022). People with different perspectives might disagree what the specific legitimate goal is or how reaching one of the rather vague goals such as well-being, development of capabilities etc. looks like to them. Even if we assume that people confronted with a medicalized phenomenon share strategic goals, there may be disagreement about whether using medical expertise and resources is a good strategy, let alone the best one, for achieving these goals.

While examples such as the medicalization of birth control where most people would agree that it is entirely clear that medical means can provide substantial benefit and that those who can get pregnant even face a certain reliance on the medical profession to live a life with all potentials (Prince 2023) this is not in every case as consensual. There are numerous controversial conditions where it is unclear whether they are well located in medicine (Kukla 2014, 2022; Wardrope 2014) and these cases are those that are often discussed as potentially harmful and oppressive.

Using the case study of medically intervening on PMS and PMDD, I will demonstrate the limitations of pragmatic analysis tools, such as Kaczmarek’s, in controversial cases. Assessing whether a medicalization is good requires a more fine-grained approach that analyzes the usefulness of medicalization in an abstract sense, as well as the usefulness of existing medical practices and their scientific basis, which may not live up to our expectations.

## PMS and PMDD – a case of controversies

Before delving into the challenges of evaluating controversial cases of medicalization with pragmatic approaches, an introduction to the case study is needed. PMS is currently understood to be a cluster of over 300 physical and behavioral symptoms disrupting everyday life experienced by people who menstruate during the luteal phase. The prevalence of PMS in the population is unknown due to unclear diagnostic criteria. Some estimates categorize any occurrence of premenstrual mood change or physical symptoms as a case of PMS, assuming that this affects around 80% of the menstruating population (King 2025). However, stricter criteria tend to yield a figure of around 20 to 30%.

While the idea that the female body is inherently problematic, both physically and psychologically, dates back to Hippocratic medicine, modern medical research has only begun to address the physical and behavioral symptoms of the luteal phase in the 1930s. This was catalyzed by advancements in endocrine science and the identification of female sex hormones. With the discovery of estrogen and progesterone, a new theory emerged, suggesting that these hormones and their effects could be responsible for such problems (Rodin 1992). Whereas in the 1930 s to 1950 s research covered both physical symptoms and behavioral symptoms, after the Second World War there was an increasing focus on behavioral symptoms that continues to this day. Both in contemporary medical, educational materials (e.g. Royal College of Obstetricians & Gyneacologists 2018) but also in societal and pop-cultural debates, the mood-based and behavioral symptoms associated with PMS, such as anger and depression, are the main focus.

Although PMS is a rather vague syndrome, the publication of the fifth edition of the Diagnostic and Statistical Manual of Mental Disorders (DSM-5, APA 2013) officially introduced a psychiatric sister-diagnosis that was already critically discussed for years: premenstrual dysphoric disorder (PMDD)^7^. The DSM-5 lists the following symptoms of the depressive disorder PMDD: a marked depressed mood, anxiety, “affective liability,” irritability/anger, a change in appetite, reduced interest in usual activities, hypersomnia or insomnia, difficulty concentrating, a sense of “out of control, " loss of energy, and physical symptoms such as breast tenderness, bloating, and weight gain (DSM-5, APA 2013). Being diagnosed requires the presence of five or more symptoms during the luteal phase, and those symptoms must diminish within a few days of the beginning of menstruation. The first-choice treatments for the mood-based and behavioral symptoms of both PMS and PMDD are pharmacological^8^. For the treatment of PMDD, some combined oral contraceptives are officially approved, and SSRIs are another primary and approved option.

PMS and PMDD are, as a matter of fact, medicalized conditions. They are medically diagnosed (at least in the case of PMDD), researched, and treated. Nevertheless, there is still controversy about whether this medicalization is appropriate (Chrisler and Gorman 2015). Evaluating the case based on the first two questions from Kazcmarek reveals how difficult it is to reach a judgment.

Intuitively, most people would probably answer “yes” to Kaczmarek’s first question, which asks whether the phenomenon of interest increases the risk of considerable physical or mental discomfort, suffering, impairment, or death. One can see that the physical and behavioral symptoms of PMS and PMDD are typically considered discomforting, or as causes of suffering. However, this judgement might also differ from person to person, from culture to culture, depending on their overall context of living, their view on the menstrual cycle in general, and their understanding of suffering (Chrisler and Gorman 2015). While many people experiencing PMS symptoms are likely to welcome medicalization based on arguments of suffering, there are also people who menstruate who describe their cycle as a “superpower”, understanding premenstrual symptoms as signs to calm down and invest in selfcare (Ussher et al. 2014). Based on these considerations, one might imagine that people who find PMS symptoms distressing are more inclined toward medicalization than those who view the symptoms as an indicator to practice self-care. The latter group may fear that medicalization goes hand in hand with shaming self-care and slowing down. They may also fear that it reinforces the demand for constant productivity and cheerfulness. Therefore the first question has potential to show disagreement and controversy over whether using medical expertise and resources is a good solution.

The second question asks about social expectations or the potentially problematic motivations and oppressive aspects of medicalization that might conflict with our broad pragmatic goals, indicating that medicine might not be the best solution to a problem. It is not intended to introduce a social-medical distinction, as medicalization based on social expectations is not necessarily a problem with a pragmatic approach. The primary concern here is whether the social expectations that support medicalization are well-founded, free from sexist, racist, ableist, or other oppressive norms and *authentically shared* by those who are medicalized. However, I will show that answering the question of whether the desire to control PMS and PMDD symptoms, especially behavioral ones, is based on oppressive norms or *exaggerated* social expectations is highly complex. Answering this question requires multiple normative and empirical components which are controversial and often unknown.

A first broad normative reflection could consider “what we want to be able to do or what should we be able to do”. Applied to the mental distress associated with PMS we could ask “do we want to control or should we be able to control our emotions in the luteal phase?” It can be said quite clearly that regardless of whether we have female or male reproductive organs, we want to be able to control our emotions to some extent. We tend to think that feeling sad when experiencing a loss of a loved one or feeling happy when getting good news are perfectly appropriate responses. Feeling very angry “out of nowhere” or simply breaking down sobbing without being able to give a real reason for this outburst, on the other hand, is something that most of us would probably not want to experience. Apart from the fact that it is simply unpleasant, it also creates potential problems in our personal and professional lives. Therefore, not being able to control one’s emotions in the luteal phase could indeed be considered a problem and not an exaggerated social expectation. So, if someone had this tendency, and a medical solution would provide relief and create the emotional stability that we generally desire, then this initially seems unproblematic. Now, with PMS and PMDD it the crucial and controversial question is exactly whether the emotional distress is “coming out of nowhere” or whether there are valid reasons for the emotional distress of menstruating people in the luteal phase.

Generally people who menstruate have reason to be skeptical about medicalization concerning their reproductive functions as historical observations indicate that female bodies have consistently been a target of medical intervention based on social expectations and for social control. Examples of this phenomenon include the attribution of hysteria for a wide range of non-conformist behaviors throughout history, as well as the treatment of menopause, which was often employed to maintain a sense of “femininity”. If the emotional distress of menstruating people has valid reasons and our wish to remove this distress would be rooted for example in harmful, sexist norms one could argue that medicalization does not promote legitimate goals.

However, a history of sexist bias in women’s healthcare alone is not sufficient grounds to suspect oppressive norms in a specific case of controversial medicalization. While women’s health has been a target of sexist bias, it has also long been characterized by a lack of research and care in some reproductive contexts such as endometriosis and in non-reproductive contexts e.g. the diagnosis of myocardial infarction in women. Therefore, medicalization can also be seen as a form of compensation for previous neglect.

In fact, one of the most controversial questions at the heart of the debate is whether the treatment of PMS and PMDD amounts to a long-overdue recognition of suffering and the provision of appropriate support, or whether it instead constitutes a sexist normalization of behavior through medication. Those promoting the inclusion of PMDD to the DSM-5 argued that the concerns of menstruating individuals have been marginalized for too long, and medicalizing the symptoms of the luteal phase prevents those affected from being told their problems are “all in their head” (Hartlage et al. 2014). Opponents of the medicalization of PMS and PMDD on the other hand argue that the medicalization of PMS and PMDD is influenced by harmful norms of emotional self-control. They hint at gender roles that emphasize the importance of self-discipline to be a good women and mother, always caring, soft-spoken, kind and patient (Chrisler & Johnston-Robledo 2002; Browne 2014), women’s subordination in the workplace, and the double burden of caregiving and employment (Chrisler et al. 2006; Ussher et al. 2014; Browne 2014) as potential reasons for very valid outbreaks. This hypothesis is supposedly supported by research showing that individuals reporting stressful living conditions, a perfectionist attitude, and a history of depression exhibited higher levels of premenstrual distress while individuals reporting greater relational satisfaction, including receiving more support and understanding from their partner during this phase, exhibited lower levels of premenstrual distress (Ussher et al. 2014; Rosvall and Ekholm 2016).

Unachievable high demands in everyday life combined with norms of emotional self-control condemning every deviation from gender roles can indeed be considered an exaggerated social expectation. Examining the initial advertisements for Sarafem, the rebranded Prozac for treating PMDD, also suggests that any display of anger or irritability in menstruating individuals is considered problematic. In one commercial, a woman gets upset when she can’t pull a shopping cart out of a row. In another, a woman searches for her keys in a stressed manner and impatiently calls out to a family member to ask if they have them. The commercial ends with a voice stating: “It’s that week before your period…the irritability, mood swings, bloating. Think it’s PMS? Think again! It could be PMDD.” If this advertisement reflects the way PMS and PMDD are actually conceptualized as problems, then skepticism seems appropriate. as We can assume that people who menstruate, whether they are in the luteal phase or not, and people who do not menstruate at all, can all react with stress when running late and being unable to find their keys or when annoyed after a stressful day because the shopping cart gets stuck (Chrisler and Gorman 2015). Yet, we only offer medical solutions to one of these groups.

However, in the context of actual treatment of PMDD the problems experienced by individuals diagnosed with PMDD differ from those shown in advertisements. These patients don’t report mood-based changes connected to stressful everyday life events such as displayed in the Sarafem advertisement. They do emphasize that mood changes come “all of the sudden"and describe their experience stating things like “I could never see any outside things, like things had happened that made me upset or made me dark” (Osborn et al. 2020). Critics argue that gender-coded behavioral expectations are so deeply ingrained in our culture, it is questionable whether those affected recognize the triggers themselves or have internalized unattainable expectations to such an extent that they have the impression that an emotional response comes out of nowhere. However, this ultimately raises the question of how we should bring together structural observations and all kinds of testimony, and how much credibility we should give to existing testimonies that claim symptoms “came out of nowhere.” Overemphasizing these testimonies could negate structural causes, and interpreting them as the internalization of harmful norms could negate lived experiences and constitute testimonial injustice (Wardrope 2014).

Evidence suggests that exaggerated social expectations play a role in the context of PMS and PMDD. But it remains unclear whether they are the main driver of our conceptualization of PMS and PMDD as problems that need *medical* treatment. We lack clear criteria to judge whether our understanding of a phenomenon as problem that requires medicalization is solely based on these expectations. Even if we could clearly show that the concern over PMS and PMDD symptoms is mainly due to problematic social expectations, some people would still consider conforming to societal norms a valid goal and medicalization a legitimate way to achieve it. Others would disagree. Therefore, Kaczmarek’s second question again can only help to make societal controversies and problems visible. However, there is no clear strategy to guide us in evaluating conflicting values, views and testimonies.

This brings us to the third and fourth questions of Kaczmarek’s pragmatic approach: whether medical resources can help us to explain and/or treat a phenomenon. According to Kaczmarek, these two questions should be addressed with the help of scientific investigation. While biomedical science can provide important insights, it may not be sufficient to offer a complete picture. She argues that other disciplines can also contribute to understanding the various potential causes of X. Although identifying the cause of X is not always necessary, it can be highly valuable in determining the most effective and safest ways to treat or resolve it.

In the case of PMS and PMDD, however, analyzing the causes and solutions again primarily reveals controversies and gaps in our knowledge. The historical analysis of PMS research by Knaapen and Weisz (2008) reveals the difficulties of biomedical science to identify the causal mechanisms behind PMS symptoms. In the 1950 s French, British and American scientists assumed that the cause of the symptoms was a hormone imbalance, e.g. an excess of estrogen, but the details of the hormone imbalance theory varied. In Great Britain, physician Katharina Dalton, who determined the early etiology and treatment, suggested a lack of progesterone as the cause of the symptoms and provided hormone replacement therapy with natural progesterone as a treatment. Other British scientists disagreed with Dalton’s theories based on the observation that psychological symptoms of PMS responded to muscle relaxants or tranquilizers (Swyer 1955; Appleby 1960; Coppen and Kessel 1963). Later studies demonstrated that progesterone therapy was as effective as a placebo. Nonetheless, the assumption that the mental distress of PMS and PMDD must be due to hormonal fluctuations or deficiencies persists in medical research and practice (King 2025). However, hormone assays have never shown any difference between menstruating people with and without PMS complaints (Chrisler and Gorman 2015). To date, no direct causal link between female sex hormones and premenstrual symptoms has been established, only a correlation between the occurrence of symptoms and their circulating levels. Other physiological substances, systems, and structures that fluctuate during the menstrual cycle are rarely discussed as potentially causal for the behavioral symptoms in PMS research (King 2025).

Current beliefs about the mechanisms underlying behavioral symptoms of PMS and PMDD are largely informed by the clinical efficacy of pharmacological treatments rather than by direct mechanistic understanding. The success of combined oral contraceptives has further prompted the belief that hormonal fluctuations in the cycle are the cause of the symptoms, while the effectiveness of SSRIs has led to the hypothesis of an interaction between ovarian hormones and the serotonergic system. However, this reasoning is circular, as it derives explanations of pathophysiology primarily from the known mechanisms of action of these drugs (Chrisler and Gorman 2015). Although these treatments can alleviate dysphoric symptoms, their efficacy does not in itself validate the proposed mechanisms, as RCTs are not designed to provide mechanistic insights.

The discussion of Kaczmarek’s second question has already indicated that social explanations for the phenomenon do exist, including overly demanding gender roles and double burdens. Nevertheless, biomedical explanations for physical, emotional, or behavioral changes associated with the menstrual cycle remain dominant in medical professional and popular literature, as well as in materials aimed at those affected. Although researchers acknowledge that the causal mechanism behind PMS symptoms is unclear, information intended for the general public often suggests that a hormonal, biomedical cause exists and that only the details remain unclear.

The reason for the emphasis on biomedical explanations is probably that the phenomenon of interest is already so heavily influenced by the existing medicalization. In disciplinary thinking, where each science has a conceptual framework for what is part of its field of interest, as is often the case in modern science, there is often no room or expertise to assess alternative explanations from other disciplines. Medical scientists focus on biomedical explanations and solutions. The isolated biomedical perspective is demonstrated by the four meetings of the International Society for Premenstrual Disorders (ISPMD) between 2007 and 2016. Neither critical PMS scholars were invited, nor was the available research on established psychosocial determinants of premenstrual symptoms integrated (King 2025). There is also no higher authority that provides standards for comparing and evaluating such at least partially competing explanations from different science in terms of their plausibility. Therefore, we cannot clearly say whether the focus on biomedical resources is more plausible for the explanation of PMS and PMDD.

We can conclude that the focus is on biomedical explanations, but that alternative explanations also exist. It is unclear to what extent these explanations may be equally plausible or even intertwined. Therefore, attempting to answer Kaczmarek’s third question, again, primarily highlights uncertainties and the existence of different perspectives.

Similar things can be said about the fourth, and probably most pragmatic question asking whether medicalizing X ensures the most effective and safest methods of solving it. As already described the primary treatment for PMS and PMDD is pharmacological, especially via combined oral contraceptives containing drospirenone and low level of estrogen or SSRIs. One could argue that success in clinical trials and approval prove success of medical resources to treat the phenomenon. However, such a conclusion is not necessarily warranted when one considers the social-epistemological structures behind these findings.

Approval of a drug does not imply having the most effective and safest method of treatment all things considered. For example, for FDA approval, in addition to Phase I and II data, only two large RCTs need to show a beneficial effect when compared to placebo, regardless of how many RCTs were actually conducted (Stegenga 2016). If unsuccessful trials had to be included, the interventions would often look significantly less successful, as in the case of SSRIs and combined oral contraceptives for PMDD. A Cochrane Review found that only two of the five trials of the oral contraceptive that it evaluated showed positive effects for women diagnosed with PMDD compared with placebo (although the placebo effect was also quite large) (Lopez et al. 2012). A 2008 review of 16 trials of SSRI and combined oral contraceptives (COCs) for PMDD found that the percentage of women who did not respond to SSRIs or COCs was actually higher than the percentage of women who did respond (Halbreich 2008).

Furthermore, we simply don’t know whether alternative interventions, for example social interventions such as changed social expectations towards people with menstrual cycles, more shared burdens of care work etc., would be more effective. Research funds are limited, and once a condition is medicalized, research on solutions is typically done within a medical framework. It is not the case that the same amount of effort is put into research on social interventions at the same time. Since much of the research in the medical field is conducted by or at least associated with the pharmaceutical industry, it can be assumed that the interventions developed reflect the interests of the industry to sell. Commercial interests do not necessarily lead to fraud or questionable study designs, but they can lead to an epistemic situation in which the body of available evidence used to make health policy decisions, and probably also treatment decisions have blind spots that distort our perceptions of the feasibility, benefits, and risks of various alternative courses of action (Jukola 2021).

But even if we were able to invest the same amount of resources in researching social interventions, there are major practical constrains. Most people living in today’s societies were socialized with specific gender roles and a medicalized understanding of premenstrual distress. It is probably impossible to create an intervention group from these people which is supposed to receive the intervention of a “just society”, as it is not possible to control for prior social experience and completely block out these influences during a study. It is also not possible to blind such a hypothetical trial. Since we apply the epistemic criteria of evidence-based medicine to medicalized phenomena and consider RCTs and their meta-analyses the gold standard, it would be difficult to attribute credibility to such a potentially biased study, even if it were technically feasible.

It thus becomes clear that the already existing medicalization is primarily self-confirming through research priorities and prevailing evidence standards and that alternative explanations and solutions remain systematically under-researched. Although we may have doubts based on the unclear mechanistic basis of PMS and PMDD or potential industry bias in drug research, we cannot conclude that there are *better* alternatives in terms of explanations and interventions.

## Learning from controversies & evaluating medicalization on different levels

Using Kaczmarek’s criteria could not determine whether intervening on PMS and PMDD with medical resources is good or bad. Answering her questions mainly revealed the existing medicalization of PMS and PMDD (which was already acknowledged to exist) and controversies surrounding the question whether medicalization can and does promote legitimate goals as described by Kukla (2022). The first controversy focuses on the question whether medicine can alleviate the suffering associated with premenstrual symptoms or if it merely hinders effective solutions, so whether it can serve shared goals at all. The other central controversy concerns whether those affected have valid reasons for their symptoms that are not rooted in the individuals themselves and their physiology and whether the environment has exaggerated expectations of how those affected should function. If the latter would be the case it would be controversial whether medically supporting conforming to these exaggerated norms would serve legitimate goals.

This suggests that the debate on good versus bad medicalization is even more complex and multifaceted than already anticipated. It raises the questions: How should we deal with these controversies, and what conclusions can we draw about controversial cases from a pragmatic standpoint? Should PMS and PMDD be medicalized—intervened on by medical professionals using medical and pharmaceutical treatments—or should de-medicalization efforts be pursued? What kind of approach is needed to understand and mitigate the potential harms of controversial cases of medicalization?

Withdrawing medical resources, or de-medicalizing, does not seem to be an appropriate way to address the controversial medicalization of issues such as PMS and PMDD. While it would demonstrate a specific stance on the first controversy, it would not necessarily lead to better practical solutions and outcomes. As previously mentioned, the process of medicalization often involves a narrow focus on medical solutions, creating ignorance of alternatives. If different actors with different kinds of knowledge and tools would be able to develop interventions with better outcomes or at least less harmful consequences, then de-medicalization might be warranted. However, the question of whether such interventions exist is precisely one of the issues or unknowns at the heart of the controversy surrounding PMS and PMDD and many other controversial cases.

It is unclear whether shifting the responsibility to minimize the suffering described by those affected by PMS and PMDD to other actors would be more promising and have fewer negative effects. Although societal explanations suggest that social changes could affect the experience of PMS symptoms, particularly behavioral ones, and that other stakeholders (e.g., society as a whole, employers, and partners) should be held accountable, there is no clear concept or evidence of successful large-scale societal interventions for PMS and PMDD symptoms^9^. Medical interventions seem to effectively manage symptoms for quite some individuals in the existing situation. Some people affected, especially those with PMDD, welcome medicalization. They feel understood, validated, cared for, and report that being recognized and treated by medical professionals changed their lives for the better (Osborn et al. 2020). Taking responsibility for reducing the suffering of those affected away from medicine—an institution that has been at least partially successful—and transferring it to other actors, without conclusive evidence that this would be the better option, does not necessarily appear less harmful.

Additionally, it is unclear if de-medicalization would benefit those who fear medicalization promotes illegitimate goals and values. Social change is often slow and depends on complex societal and political processes that are difficult to direct and predict. They can be influenced by the same problematic value judgments and exaggerated social expectations suspected to be at play in medicalization. Often, it is not medicalization *qua* medicalization that generates problematic outcomes, but *rather the specific value-laden assumptions attached to a particular phenomenon that become operative within a medical context*—shaping how medicalization is implemented and communicated. The normative influences that inform how we conceptualize, operationalize, and address certain phenomena in medicine may travel, and produce similar concerns, even if responsibility for these phenomena is shifted to other actors, such as policymakers or social institutions.

The withdrawal of medical solutions does not automatically result in the immediate availability of social alternatives or in the elimination of oppressive social norms. Instead, it could leave those who welcome medicalization without adequate means to address their suffering for an extended period of time or certainty that there will be an alternative in the future. Therefore, de-medicalizing controversial cases such as PMS and PMDD cannot be a satisfactory solution. Instead, we need strategies that address the different views and needs of people and open up spaces to discuss the improvement of existing solutions and the development of alternatives.

In order to constructively discuss the goods and bads, or the appropriateness of specific cases of medicalization, it is necessary to gain a more precise understanding of the expectations underlying the controversies. Understanding these expectations can help us reflect openly on the extent to which they are or are not being met, on whether and how existing medicalization can be improved, and on how the door can be opened to de-medicalization. While I don’t assume that this analysis and subsequent discourse will find the best solution for everyone, they give us the opportunity to move beyond merely recognizing controversy and improve the status quo. It can also reduce the pressure to accept certain solutions as inevitable, opening doors to alternatives.

I suggest that the controversies are rooted in at least two fundamental expectations about the goodness of medicalization: one more abstract and the other more concrete. The first and more abstract expectation is that medical expertise and resources have to be potentially useful for achieving our goals. Here, goodness as usefulness aligns with a pragmatic approach that considers the key factor in deciding whether to intervene on a condition medically to be whether medicine, given its role, expertise, and available tools, can achieve broader goals, such as individual or collective well-being and the development of capabilities.

However, the actual usefulness of medicine may differ from its potential usefulness. A view on the history of medicine and its scientific advances shows that complete knowledge is rarely available and that knowledge may not be sufficient to provide solutions to a problem, just yet. Nevertheless, we often leave such questions and phenomena to the realm of medicine, since this is where we anticipate seeing progress or finding other useful resources. As medicine is constantly evolving and effective solutions often require years of persistence, it is sensible to consider in a first step whether there is any potential usefulness in having the kind of knowledge medicine produces, the kind of social relationship the doctor-patient relationship offers and the virtues that are deeply institutionalized in medicine. If human or epistemic resources, or the tools of medicine, are not even useful in the abstract sense, medicalization can easily be critized and the de-medicalisation of a phenomenon can likely be justified by referring to this deficiency. If people have very different needs when confronted with the medicalized phenomenon, and if medicalization can be beneficial for some but not for others, then this should be made transparent. Doing so would help us better understand the differences between various groups and strengthen the legitimacy of non-medical approaches.

If we conclude that medicalizing a condition is, in principle, beneficial and at least not inherently harmful or oppressive, the particular ways in which medicalization is implemented should be evaluated in the second step. While medicalization may be the best available solution right now, in an abstract sense, based on the potential usefulness of medical epistemic resources and tools, as well as professional virtues, the concrete communication, conceptualization, operationalization, and use of these resources and tools can be restrictive, misleading, and stigmatizing nonetheless. A good example of this is the medicalization of birth control. Medical, epistemic, and social resources are ideal for developing interventions and providing support and advice on their use. We probably wouldn’t want other professionals to be concerned with these matters. However, the way the benefits of contraceptives are often conceptualized as epidemiological goals, such as reducing female mortality, while conceptualizing side effects, such as libido loss, as minor raises controversy as it marginalizes the fundamental interests and intent of people who use contraceptives to have enjoyable sex (Prince 2023). Historically, and to this day, people who can become pregnant are conceptualized as passive participants in sexual encounters who need to be protected from the consequences. These conceptualizations do not only misrepresent the motivation of users to use medicine it also creates obstacles to needed side effect information (Prince 2025).

The second, more concrete expectation underlying medicalization controversies seems to be that, if goodness can be identified as usefulness in an abstract sense, existing medicalization should realize or, at the very least, not contradict this usefulness. Clearly, there will probably be a lot of disagreement about what legitimate goals we want to pursue by medicalizing something and it is unlikely to resolve them. At the very least, however, we can assume that implementing medicalization in a way that only contradicts the fundamental user intention or conflicts with our fundamental reasons to medicalize something, as identified in step one, cannot be legitimate. The second step naturally builds on information from the first step. While the first step assesses whether medicine has helpful social practices, epistemic resources, and tools, it also aims to illuminate the societal (and individual) expectations that arise from this usefulness. The second step of an analysis of medicalization should examine whether the practical implementation meets or contradicts these expectations.

Understanding the specific problematic aspects of medicalization at different levels in our particular cases allows us articulate more precisely in what respects medicalization is good and in what respects it isn’t. A medicalization that can’t show any, not even potential usefulness should be given up, while a medicalization that is useful to some but not to others would benefit from discussions on whether we have a proper understanding of the phenomenon and those affected. In other cases we might see big potential for medicalization but the actual way it is implemented is harmful. Understanding these complexities allows us to address these issues and identify possible countermeasures that help to realize the abstract usefulness of medicalization and to preserve medicalization for those who value it while minimizing the associated harms.

## The good and bad of medicalizing PMS and PMDD at different levels

The following section will demonstrate that a two-step, pragmatic analysis tool is capable of going beyond highlighting controversies and providing a more detailed description of the problems associated with the medicalization of PMS and PMDD, as well as developing solutions. Due to space limitations, I will not be able to conduct a full reanalysis of the PMS/PMDD case. In the first step, I will present arguments supporting the position that, despite the controversies, we have pragmatic reasons to intervene on PMS and PMDD medically because of the abstract usefulness of this approach given the nature of these phenomena. In the second step, I will reflect on real-life practices of PMS/PMDD medicalization in light of the identified aspects of usefulness.

Recall that, according to the first pragmatist analysis step, the key factor in deciding whether to intervene on a condition medically is whether medicine, given its role, expertise, and available tools, is useful in achieving our broader goals. The analysis based on Kaczmareks questions identified controversy over whether the medical hypotheses concerning the causes of PMS and PMDD are fully convincing. Does this mean that medical knowledge and hypothesizing are not useful for understanding PMS or PMDD? Considering the known characteristics of our phenomenon of interest implies that we can assign medical knowledge at least potential usefulness. While the exact causal mechanisms behind PMS and PMDD symptoms are unknown, the symptoms do correlate with the physiological processes of the female cycle. Understanding physiological processes and intervening on them is undoubtedly a significant aspect of medical expertise and responsibility (Sackett et al. 1996). In this respect, medical inquiry clearly serves a relevant interest, to gain understanding, at least partially. The fact that we don’t yet have a proper understanding of a phenomenon, or that it may require a complex, multifactorial explanation, does not indicate that medical epistemic resources are not helpful or necessary in achieving this understanding.

Regarding the solutions developed through medical and pharmaceutical research, it can be said that they are considered helpful by some of those affected, at least. Therefore, it can be assumed that medicine can help some affected to achieve their goals. Even if medical treatments for PMS and PMDD only help people to cope with society’s exaggerated expectations (which is debated), this is consistent with other accepted medical treatments. For example, psychotropic drugs are often prescribed as a first step (with additional psychotherapy) to help people cope with stressful circumstances while they try to change them. However, it is important to acknowledge the pragmatic nature of this kind of usefulness, which does not imply that there is something wrong with the person or their physiology. Furthermore, it is important to carefully consider the scope of the usefulness of interventions. The discussion based on Kaczmarek’s questions revealed that there is no consensus on the usefulness of interventions that merely enable people to adapt to problematic societal norms. Therefore, in a second step, particular attention should be paid to how the different needs and views of those affected regarding usefulness are dealt with.

Another aspect speaking in favor of a pragmatic medicalization of PMS and PMDD is the social role of doctors in society. Society grants the medical profession prestige, substantial rewards, autonomy in practice, and the privilege of self-regulation. In return, the profession is expected to act with honesty and integrity, prioritize the interests of patients and society over their own interests, address societal concerns within their domain, and ensure the competence of practicing physicians (Cruess & Cruess 2020). Consequently, people hold a variety of expectations of physicians that go beyond science-based medical knowledge, accurate diagnosis, and appropriate treatment. Because communication of personal information and physical examinations require so much trust and put patients in vulnerable positions, doctors are also required to provide a certain extent of social care. They do justice to the patient’s vulnerability by following institutionalized rules, such as a commitment to confidentiality as part of the professional ethos (Rhodes 2020). Furthermore, the medical profession adheres to more virtues that are not as clearly institutionalized. One such virtue is having a caring and empathetic attitude toward patients and communicating clearly and truthfully (Pellegrino 2002; Hubert et al. 2020; Brenner et al. 2021; Desai & Kapadia 2022). This form of social care, which includes empathy, collaboration, and reassurance in difficult times, is a vital part of the profession and something that people increasingly struggle to find in other institutions nowadays.

Literature dealing with the role of empathy and care in the doctor-patient relationship suggests that empathy-based interactions between patients’ emotional vulnerability and doctors’ emotional availability not only increase patient autonomy, but can also positively influence patients’ physiological state (e.g. stress response) (Adler 2002; Neumann et al. 2009). In the absence of alternative accessible institutions that provide social care, and given the expressed need of those affected to feel understood, validated and cared for, doctors, through their professional norms and virtues, seem capable of addressing some aspects of the distress and vulnerabilities associated with PMS and PMDD. This is regardless of whether we can attribute PMS and PMDD to social causes, or whether we believe that social changes could be more effective in addressing the problem. However, it can’t be stressed enough that this pragmatic usefulness stems from the way we organize our societal institutions and the virtues and practices found within them. In theory, we could organize society in a way that provides these virtues and social practices within a different institution.

Regarding our first step of the analysis one can conclude that for PMS and PMDD medicine seems to be able to offer at least the best preliminary solution by holding relevant epistemic resources for understanding the phenomenon, providing a relevant caring relationship^10^, and interventions that might be imperfect but helpful for some.

Now, in the second step, I will highlight conflicts between reasonable expectations that one might have based on the usefulness of medical epistemic resources, the professional ethos of the discipline and the actual real-life practices (research, clinic and communication) we can observe in the medicalization of PMS and PMDD. I already hinted at some things to be cautious about and the importance of this caution will get become more clear when discussing the actual ways PMS and PMDD are medicalized.

The first and primary problematic practice that potentially contradicts general norms of medicine and society’s expectations of medicine is the tendency to conceptualize PMS and PMDD as a primarily hormonal phenomenon without adequate justification. As previously mentioned, the mechanistic hypotheses proposed to explain the relationship between sex hormones and premenstrual symptoms have not been confirmed. Nonetheless, educational material, research, and doctor-patient communication often attribute any physical, emotional, or behavioral changes associated with the cycle to hormonal changes or even unbalanced hormones (Chrisler and Gorman 2015). This approach not only disregards the ongoing discussion about social factors that influence the experience of premenstrual symptoms, but it also ignores additional, existing physiological knowledge about the menstrual cycle.

King (2025) convincingly argued that multiple other cyclical fluctuations beyond the female sex hormones (such as water retention, iron levels etc.) are part of the menstrual cycle, which is not only a hormonal process but also an inflammatory immune response. However, those phenomena have hardly been researched in terms of their possible connection to premenstrual symptoms (Chrisler and Gorman 2015; King 2025). This is surprising, given that raised levels of certain inflammatory markers have been found to be associated with most common premenstrual symptoms. They constitute one of the few statistically significant ‘biological’ differences ever found between people experiencing PMS and those who do not (Bertone-Johnson 2016; Bertone-Johnson et al. 2014; Gold et al. 2016). Inflammation is also believed to be linked to the bidirectional relationship between psychosocial experiences and biological changes. External life stressors directly impact the immune system by increasing the number of circulating inflammatory cells and causing it to produce ‘excessive inflammatory responses’. A chronically activated immune system can also negatively impact a person’s mental health, particularly their mood (King 2025).

Suggesting a causal link between female hormones and distressing symptoms is not necessarily “wrong” or sexist (King 2020). However, such conceptualizations often perpetuate the false assumption that if distressing symptoms are caused by fluctuating female hormones, then everyone with corresponding hormones experiences those symptoms. These kinds of fallacies perpetuate myths about women’s “sensitivities” and “irrationalities.” Given the lack of scientific evidence supporting hormonal explanations for premenstrual symptoms, it is understandable that the legitimacy of medicalization goals is being questioned. Focusing solely on hormonal explanations and ignoring other potential physiological explanations can create the impression that medicine is not utilizing all existing physiological knowledge about the cycle. Instead, it appears that medicine is guided by social stereotypes in its conceptualizations, which form the basis for explanations and solutions. This would conflict with general expectations of medicine as a science-based discipline, as it ignores relevant evidence and allows non-epistemic values to potentially distort inquiry.

The hormonal conceptualization of PMS and PMDD does not only influence the available knowledge it is also very present in the ways information about PMS and PMDD are described and communicated in public (e.g., health information online) and in clinical patient information. Even if it is acknowledged that there is no full clarity, it is communicated as though it is known that the causes are hormonal, while only the details are unclear. Communicating this way without acknowledging the rather big uncertainties around the causes of premenstrual symptoms and the existence of social explanations conflicts with principle of honest, truth-based communication aimed at optimal understanding. Furthermore, the use of discursive resources (words, concepts, and expressive styles) that e.g., locate and describe phenomena in the individual, etc. has impact on how those affected can describe their own experiences and on how solutions can be discussed. Influenced by the authority of the discipline, patients might tend to accept individual physiological explanations as given and may gain the impression that a medical solution to a problem is inevitable.

Here is where the pitfall of entrusting a profession with certain tasks for pragmatic reasons. based on the assumption that it has the appropriate expertise, social relationships and authority, becomes apparent. We rarely see an appropriate contextualization of the role of the doctor or the entire discipline when it is applied pragmatically. Patients may not realize that medicine is only one option currently available for a problem that could be understood and addressed in additional or different ways. This impression is reinforced by the fact that it is rarely clearly communicated by physicians that it is indeed the case that they also carry out activities to give their patients the feeling of validation, and to convey care broadly understood. These activities, which are perfectly justifiable under a pragmatic approach to medicine, don’t necessarily imply that there is something wrong with the patient’s body or that certain malfunctions of the body need to be corrected. But assuming and communicating a physiological-hormonal problem that is best treated pharmaceutically implies, especially if one does not communicate that medicine does more than combat dysfunction, precisely that the problem of mental distress in PMS and PMDD can *only* be a physiological problem and exerts corresponding pressure to use medication. Neither the epistemic problems behind this explanation nor the existence of further explanations and potential alternative solutions are dealt with transparently.

The analysis presented shows that, although the medical profession is potentially well-suited to intervene on and treat certain phenomena, successfully implementing medicalization poses major challenges. Assuming that a pragmatic view of the usefulness of medicine automatically leads to fewer problems or challenges would be misguided. However, the two-step analysis outlined above makes it possible to clearly articulate expectations and critically evaluate implementation. In the case of PMS and PMDD, the problem appears to lie in how medicalization is carried out, rather than in the fact that it takes place. Offering an imperfect medical solution to a problem whose basis is potentially multifactorial and not well understood can be unproblematic. There is no problem with medicalizing a person who has mental health problems in the luteal phase if they themselves have no other way of resolving those problems at that time and find it helpful. However, the way it is done in the case of PMS and PMDD makes it difficult for sufferers to find other solutions or identify causes of their symptoms in their own lives and circumstances. When it comes to the medicalization of these phenomena, it’s crucial to address uncertainties, knowledge gaps, and the social care aspect of medicine with transparency. Of course, being more informed about the pragmatic nature of certain medicalizations does not free people from social pressure to conform, but it does give them the opportunity to share ideas, choose not to accept problematic norms, and find like-minded people who do the same. What we should not do in these controversial cases, is tell patients and society as a whole that a medical approach is the only explanation or solution to the problem. Only by being transparent about our already pragmatic approach to medicine in practice, we can help and open the door to the necessary societal debates.

## Conclusion

Taking a pragmatic approach to medical concepts and interventions helps avoid many of the problems associated with strictly defining the boundaries of medicine. This approach considers the complex interplay between social, political, physical, and mental factors that contribute to medicalized phenomena. However, pragmatic accounts also invite controversy regarding the normative basis for evaluating medicalization, which involves debating whether medicalization can and does promote legitimate goals. This makes it difficult to classify individual cases of controversial medicalization as illegitimate or even oppressive and to address these problems by removing conditions and interventions from the medical field. I argued that a two-step approach is needed to analyze whether the medicalization of a condition is good or desirable. This approach gives justice to the complexities of medicine’s abstract usefulness and difficulties in realizing this usefulness in practice. In the case of the medicalization of PMS and PMDD, this type of analysis has revealed good reasons for medicalization. However, the way it is implemented, particularly with regard to research priorities and communication, does not do justice to the phenomenon’s complexity or medicine’s abstract usefulness.

The answer to the question of the legitimacy of medicalization evolves alongside scientific progress and changes in social practices. However, providing answers will often require case-by-case assessments and a society capable of viewing and discussing the use of medicine in a nuanced manner.

## Footnotes

1 I will not be able to provide a complete analysis of the legitimacy of medicalizing PMS and PMDD.

2 Unlike Sholl, who equates intervention with treatment based on Conrad’s work, I have a broader understanding of intervention. Intervention includes treatment, which aims to reduce symptoms or their causes (i.e., healing). However, I also believe that we medically intervene and impact a patient’s well-being when we run tests, provide consultations, etc. Discussing my own account I use this broader understanding of intervention.

3 In cases of medicalization, which we understand as practices of intervention, physiological medical knowledge can play a significant role in understanding the phenomenon being intervened upon and the intervention itself. However, there is no pathologization; that is, there is no classification as a disease or pathology.

4 Of course, this harm of medicalization as a form of definition or diagnosis can have an impact on the possibilities at the intervention level. However, this is a consequence of this potential harm, not of medicalization alone.

5 I refer to medicalization based on these considerations as pragmatic medicalization.

6 It is important to note that in Kaczmareck’s account, the question regarding causes does not aim to distinguish between social and medical factors in order to differentiate between good and bad medicalization. Rather, it primarily assesses the utility of medical resources in understanding the phenomenon.

7 Previous versions of the DSM have already discussed premenstrual symptoms as potentially being of interest and requiring further research.

8 Pharmacological treatments for PMS are typically off-label, since there is no drug-testing for conditions without clear diagnostic criteria.

9 Ideas such as those put forward by Hacking (2007) regarding ‘looping effects’ and ‘making up people’ support the notion that different stakeholders would alter our societal view of PMS/PMDD, as well as the people themselves who experience these symptoms. However, it is unclear what this would look like.

10 This is especially true since those affected by PMS and PMDD often take on the caregiving role simply due to their gendered role within the family. This makes it even more difficult for them to access social care themselves.
